# Comprehensive Analysis of Stromal and Serum Markers in Gastric Cancer

**DOI:** 10.32607/actanaturae.11753

**Published:** 2022

**Authors:** O. V. Kovaleva, P. A. Podlesnaya, V. L. Chang, N. A. Ognerubov, A. N. Gratchev, N. A. Kozlov, I. S. Stilidi, N. E. Kushlinskii

**Affiliations:** N.N. Blokhin National Medical Research Center of Oncology of the Ministry of Health of Russian Federation, Moscow, 115552 Russia; Medical Institute of G.P. Derzhavin Tambov State University, Tambov, 392000 Russia

**Keywords:** gastric cancer, PD-1, PD-L1, stroma, prognosis

## Abstract

A comprehensive analysis of the cell phenotype of the inflammatory infiltrate of the tumor stroma represents a promising area of molecular oncology. The study of not only soluble forms of various immunoregulatory molecules, but also their membrane-bound forms is also considered highly relevant. We performed a comprehensive analysis of tissue and circulating forms of the PD-1 and PD-L1 proteins, as well as macrophage and B-cell markers in the tumor stroma of gastric cancer, to assess their clinical and prognostic significance. The tumor and blood plasma samples from 63 gastric cancer patients were studied using ELISA and immunohistochemistry. Malignant gastric tumors were shown to be strongly infiltrated by B-cells, and their number was comparable to that of macrophages. For PU.1 expression, an association with tumor size was observed; i.e., larger tumors were characterized by fewer PU.1+ infiltrating cells (p = 0.005). No clinical significance was found for CD20 and CD163, but their numbers were higher at earlier stages of the disease and in the absence of metastases. It was also demonstrated that the PD-L1 content in tumor cells was not associated with the clinical and morphological characteristics of GC. At the same time, PD-L1 expression in tumor stromal cells was associated with the presence of distant metastases. The analysis of the prognostic significance of all the markers studied demonstrated that CD163 was statistically significantly associated with a poor prognosis for the disease (p = 0.019). In addition, PD-L1 expression in tumor cells tended to indicate a favorable prognosis (p = 0.122). The results obtained in this work indicate that the study of soluble and tissue markers of tumor stroma is promising in prognosticating the course of GC. The search for combinations of markers seems to be highly promising, with their comprehensive analysis capable of helping personalize advanced antitumor therapy.

## INTRODUCTION

Gastric cancer (GC) is one of the most common cancers worldwide and one of the major causes of mortality. The incidence of cancer is higher in men than it is in women [1]. A large number of different factors, including Helicobacter pylori infection [2], smoking [3], dietary habits [2], genetic disorders [4], and others, lead to the appearance of GC. Although the majority of etiological factors of GC appearance are known, early diagnosis of the disease remains somewhat challenging due to its asymptomatic development, and, more often than not, the pathology is diagnosed at late stages. Combination regimens, including fluoropyrimidine and platinum drugs (and trastuzumab in the cases of HER2-positive tumors) in the first line and paclitaxel with or without ramucirumab, in the second line, are standard treatments for advanced GC. However, the median survival time in advanced GC remains approximately 12 to 15 months, obviously as we await new therapies to come on line [5, 6, 7]. Immune checkpoint inhibitors (ICIs) have recently become the new standard treatment for several malignancies, including advanced cancer. However, the success currently enjoyed with immunotherapy for GC remains limited. There are several clinical trials focusing on different combinations of immunotherapy and chemotherapy drugs to maximize efficacy. It also remains controversial whether the number of PD-L1-positive tumor cells affects the effectiveness of therapy and whether their number should be considered when prescribing an appropriate treatment. In addition, the qualitative and quantitative composition of the tumor microenvironment can affect the success of GC therapy. For example, an increased number of Th1 cells promotes inflammation and the development of cancer [8], and the content of B cells expressing IL-10 affects the production of cytokines by CD4^+^ and CD8^+^ T cells [9].

The main types of tumor immune infiltrate cells include macrophages and T-cells, as well as B-cells. It is known that the number of stromal cells and their population composition may be a prognostic factor for both the course of the disease and response to therapy. PU.1 is a transcription factor that plays an important role in hematopoiesis, and its expression at a high level is characteristic of macrophages. We have previously shown that, for various types of solid tumors, PU.1 can be used as a marker of tumor-associated macrophages [10]. CD3 is a surface marker of mature T cells and is used to determine their total content in various tissue types. CD20 is a transmembrane protein expressed on the surface of B-cell precursors and mature B-cells, allowing its use in various clinical studies as a general B-cell marker.

The purpose of this work was to perform a comprehensive analysis of PD-L1 expression in tumor and stromal GC cells, as well as the content of the soluble form of PD-L1 in the blood plasma of patients. In addition, we analyzed the content of tumor-associated macrophages and B-cells in the stroma of GC tumors.

## EXPERIMENTAL

The study included 63 primary GC patients at different stages of the tumor process and 60 healthy donors who underwent examination and treatment at the N.N. Blokhin National Medical Research Center for Oncology of the Ministry of Health of Russia. All procedures performed in the study involving patients and healthy donors met the ethical standards of the organization’s ethics committee and the 1964 Declaration of Helsinki and its subsequent amendments or comparable ethical standards. Informed consent was obtained from each of the participants included in the study. The clinical diagnosis of gastric cancer in all patients was confirmed by a morphological examination of the tumor according to the International Histological Classification of Tumors of the Digestive System (WHO, 2019). A description of the studied sample is presented in Table 1.

**Table 1:** Clinical and morphological characteristics of patients with gastric cancer

Characteristics	Number of cases, %
Age	
≤ 61	32 (51)
> 61	31 (49)
Gender	
Male	35 (56)
Female	28 (44)
Histology	
Adenocarcinoma	52 (82.5)
Signet-ring cell carcinoma	10 (16)
Undifferentiated cancer	1 (1.5)
Stage	
I–II	25 (40)
III–IV	38 (60)
Localization	
Distal	14 (22)
CEC (cardioesophageal cancer)	3 (5)
Proximal	16 (25)
Stomach body	26 (42)
Total lesion	4 (6)
Tumor size (T)	
T1–T2	13 (21)
T3–T4	50 (79)
Nodal status (N)	
N0	24 (38)
N+	39 (62)
Metastasis (M)	
M0	54 (86)
M+	9 (14)
Grade (G)	
G1–G2	19 (30)
G3	44 (70)

The concentration of sPD-L1 and sPD-1 proteins was determined in blood plasma obtained according to the standard technique before specific treatment using Human PD-L1 Platinum ELISA and Human PD-1 ELISA kits (Affimetrix, eBioscience, USA) according to the manufacturer’s instructions. Measurements were performed on a BEP 2000 Advance automated enzyme immunoassay (Siemens Healthcare Diagnostics, Germany). The protein content was expressed in picograms (pg) per 1 ml of blood plasma.

Immunohistochemical (IHC)-study of CD163, PU.1, and CD20 was performed according to the standard technique on tumor tissue sections. Tris-EDTA buffer pH 9.0 (PrimeBioMed, Russia) was used for antigen retrieval. The primary antibodies to PU.1 (4G6; PrimeBioMed, Russia, dilution 1 : 200), CD163 (10D6; BIOCARE, USA, dilution 1 : 100), and CD20 (clone PBM-12F1; PrimeBioMed, Moscow, dilution 1 : 100) were incubated for 30 min. The PrimeVision Ms/Rb HRP/DAB detection system (78-310004, PrimeBioMed, Russia) was used according to the manufacturer’s instructions.

The preparations obtained were evaluated using an OLYMPUS BX53 microscope, a Lumenera INFINITY2-2C camera, and the Infinity analyze software. The expression of CD163, PU.1, and CD20 was assessed in the tumor stroma. In each case, the number of CD163-, PU.1-, and CD20-positive cells was analyzed under ×200 magnification in five independent fields of view by direct counting. The sample was considered positive if at least one specifically stained cell was present. The content of CD163, PU.1, and CD20 in the tumor stroma was expressed as the average number of cells per field of view.

The data obtained were processed using the GraphPad Prizm 9.0 software. Mann-Whitney nonparametric test and Spearman rank correlation coefficient were used to compare the parameters and analyze their relationships. For the overall survival rate analysis, the patients were divided into two comparison groups depending on the median content of the studied proteins. The analysis of overall survival was performed by constructing survival curves according to the Kaplan-Meier method. The comparison of the statistical significance of differences was performed using the logarithmic rank criterion. To assess the potential impact of various risk factors on survival, we additionally performed a multivariate analysis using a nonparametric Cox proportional hazards model. Differences and correlations were considered statistically significant at p < 0.05.

## RESULTS

**Fig. 1:**
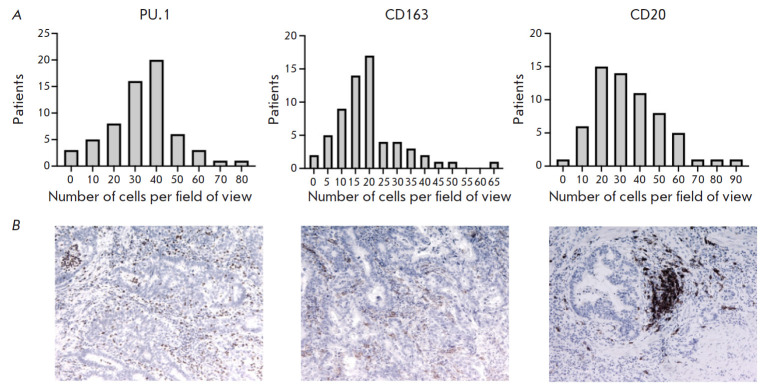
(*A*) Distribution of PU.1, CD163, and CD20 in the stroma of the tumors of GC patients. (*B*) Immunohistochemical staining of gastric tumors using antibodies to PU.1, CD163, and CD20 (×100)

Expression of PU.1, CD163, and CD20 was detected in 100% of the examined GC samples. The distribution of cell numbers in the GC samples is shown in Fig. 1.

The analysis of the results showed that the median number of PU.1+ cells in the sample was 34.8 (0.4–77.8) cells per field of view, CD163+ cells – 17.6 (0.8–66.4), CD20+ cells – 32.2 (3.2–91.2). It should be noted that, in gastric tumors, B-cells are present in similar numbers as PU.1+ macrophages.

**Association of PU.1, CD163, and CD20 content with clinical and morphological characteristics of GC **At the next stage, we analyzed the association of the PU.1+, CD20+, and CD163+ cell content in the tumor stroma with the clinical and morphological characteristics of the disease (Table 2).

**Table 2:** Association of the PU.1+, CD163+, and CD20+ cell content in the tumor stroma with the clinical and morphological characteristics of the disease

Characteristics	PU.1 (number of cells)		CD163 (number of cells)		CD20 (number of cells)	
	Median (25–75%)	P	Median (25–75%)	P	Median (25–75%)	P
Age						
≤ 61	35.8 (23.4–42.7)	0.488	17.2 (9.05–22.3)	0.297	28.2 (19.4–45.4)	0.418
> 61	34.2 (20.2–42.0)		18.2 (13.2–25.2)		34.4 (20.8–45.2)	
Gender						
Male	33.6 (20.2–37.6)	0.150	16.2 (10.4–24.4)	0.713	29.4 (18.8–45.2)	0.403
Female	37.3 (26.9–44.2)		18.0 (12.9–21.1)		33.9 (22.9–44.9)	
Histology						
Adenocarcinoma	35.0 (20.8–41.9)	0.216	17.6 (11.8–24.1)	0.459	33.3 (19.9–44.8)	0.574
Signet ring cell carcinoma	29.7 (23.3–37.7)		17.5 (10.8–19.7)		30.5 (22.1–47.8)	
Undifferentiated cancer	63.4 (63.4–63.4)		28.2 (28.2–28.2)		19.6 (19.6–19.6)	
Stage						
I–II	35.8 (27.5–44.8)	0.249	17.8 (13.7–20.7)	0.623	34.4 (21.6–46.3)	0.424
III–IV	33.8 (18.0–39.5)		16.7 (10.1–24.6)		29.4 (19.5–43.9)	
Localization						
Distal	34.8 (28.9–44.1)	0.226	17.5 (12.4–23.4)	0.824	33.8 (24.7–43.1)	0.316
CEC (cardioesophageal cancer)	35.8 (0.4–41.8)		19.4 (14.8–25.2)		23.4 (6.0–24.6)	
Proximal	33.6 (12.5–41.2)		17.3 (11.8–24.1)		28.8 (13.3–52.4)	
Stomach body	33.4 (24.4–39.8)		18.1 (11.6–23.6)		37.7 (22.3–47.3)	
Total lesion	48.4 (38.1–61.0)		11.1 (7.7–24.5)		26.5 (18.6–41.1)	
Tumor size (T)						
T1–T2	41.8 (35.5–54.4)	0.005*	17.8 (14.5–23.0)	0.504	36.0 (26.1–48.1)	0.277
T3–T4	32.9 (19.1–38.7)		17.6 (10.1–23.4)		29.4 (19.5–43.9)	
Nodal status (N)						
N0	35.5 (25.3–42.8)	0.733	17.3 (12.9–20.0)	0.437	33.3 (19.9–44.4)	0.947
N+	34.8 (20.2–41.4)		17.8 (11.4–28.2)		29.4 (20.6–45.2)	
Metastasis (M)						
M0	34.8 (25.6–41.9)	0.889	17.6 (11.2–23.4)	0.598	33.3 (21.1–45.3)	0.214
M+	35.4 (13.6–48.6)		18.4 (12.8–25.4)		29.4 (10.6–40.2)	
Grade (G)						
G1–G2	37.6 (25.0–49.8)	0.131	19.0 (13.2–25.8)	0.448	33.2 (22.0–43.6)	0.796
G3–G4	34.2 (18.7–38.9)		16.2 (10.9–21.5)		33.4 (18.6–45.4)	

^*^Statistically significant.

The analysis showed that the PU.1 content was significantly associated with tumor size; i.e., larger tumors were characterized by a smaller number of PU.1+ infiltrating cells. We should also note the differences in the content of PU.1+ and CD163+ cells, depending on tumor localization. Thus, in the case of a total gastric lesion, the highest number of PU.1+ cells and the lowest number of CD163+ cells were observed. But these observations did not reach the threshold of statistical significance.

**Table 3:** Association of PD-L1 content in tumor cells and tumor stroma with the clinical and morphological characteristics of the disease

Characteristics	PD-L1 tumor (n)			PD-L1 stroma (n)		
	+	-	P	+	-	P
Age						
≤ 61	8	24	0.117	18	14	0.609
> 61	14	17		20	11	
Gender						
Male	12	23	> 0.999	18	17	0.127
Female	10	18		20	8	
Histology						
Adenocarcinoma	19	33	0.704	32	20	0.567
Signet ring cell carcinoma	3	7		5	5	
Undifferentiated cancer	0	1		1	0	
Stage						
I–II	8	17	0.790	16	9	0.793
III–IV	14	24		22	15	
Localization						
Distal	3	11	0.396	8	6	0.987
CEC (cardioesophageal cancer)	0	3		2	1	
Proximal	7	9		10	6	
Stomach body	11	15		16	10	
Total lesion	1	3		2	2	
Tumor size (T)						
T1–T2	3	10	0.515	11	2	0.058
T3–T4	19	31		27	23	
Nodal status (N)						
N0	7	17	0.588	13	11	0.597
N+	15	24		25	14	
Metastasis (M)						
M0	21	33	0.144	36	18	0.023*
M+	1	8		2	7	
Grade (G)						
G1–G2	7	12	> 0.999	14	5	0.239
G3	12	21		18	15	

^*^Statistically significant.

**Fig. 2:**
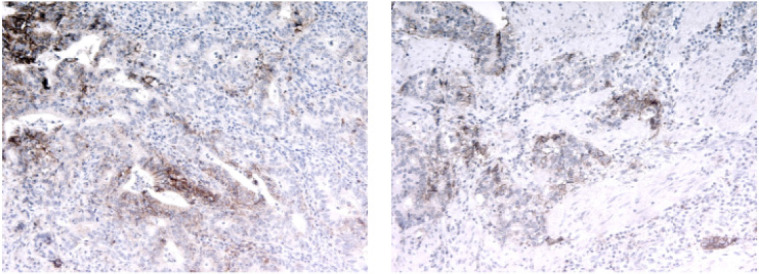
PD-L1 expression in GC samples (×100)

**PD-1 and PD-L1 content in tumor samples of GC patients **In addition to analyzing the expression of stromal markers, we assessed the tissue content of PD-L1 in the studied GC samples. Examples of immunohistochemical staining for PD-L1 are shown in Fig 2.

PD-L1 expression in the tumor cells was detected in 35% (22 of 63) of the samples. PD-L1 expression in stromal cells was detected in 60% (38 of 63) of the samples. Then, we analyzed the association of the PD-L1 content with the clinical and morphological characteristics of the disease (Table 3).

This study showed that the PD-L1 content in tumor cells had no association with the clinical and morphological characteristics of GC. PD-L1 expression in tumor stromal cells was found to be associated with the presence of distant metastases; i.e., PD-L1 expression in the primary tumor stroma was observed less frequently in their presence.

**Soluble forms of PD-1 and PD-L1 **In addition, we analyzed the content of soluble forms of the proteins (sPD-1, sPD-L1) of the immunity checkpoint PD-1/PD-L1 in the plasma of RC patients in order to attempt to identify any correlations between their content in plasma and tissue expression and prognostic significance.

At the first stage, we assessed the diagnostic potential of the studied proteins. The median sPD-1 and sPD-L1 content in the blood plasma of healthy donors was 29.25 (14.9–45.5) pg/ml and 36.23 (9.83–73.1) pg/ml, respectively; and in the group of GC patients – 12.57 (7.7–19.7) pg/ml and 21.83 (10.1–74.3) pg/ml. The statistical analysis showed that the content of the soluble form of the sPD-1 receptor was significantly lower in GC patients compared to the healthy donors. The levels of sPD-L1 did not differ between the groups of healthy donors and GC patients.

**Fig. 3:**
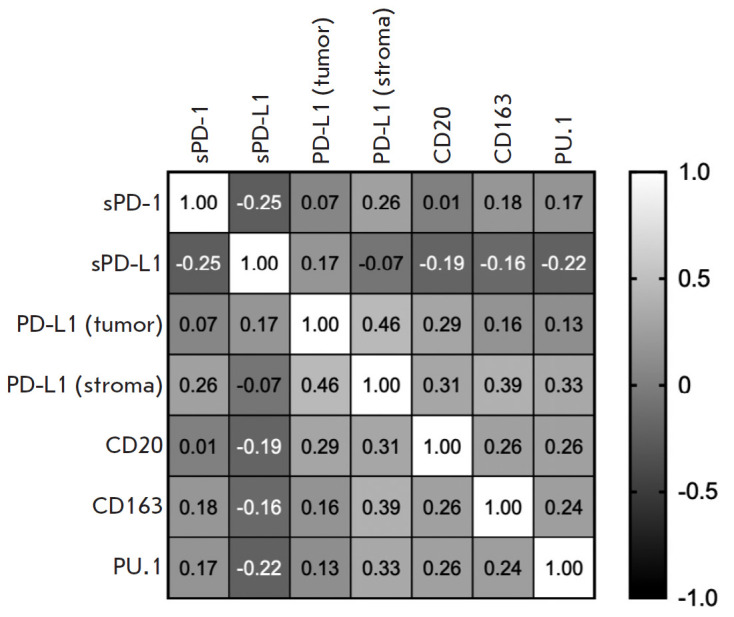
Correlation analysis between tissue and serum levels of PD-1, PD-L1, PU.1, CD163, and CD20 in gastric cancer patients

**Correlation analysis of soluble and tissue forms of the studied proteins **We performed a correlation analysis of the proteins examined by determining the Spearman rank correlation coefficient. The results are shown in Fig. 3.

The analysis showed that the plasma content of the soluble form of the sPD-1 receptor inversely correlates with the plasma content of sPD-L1 and directly correlates with the tissue expression of PD-L1 in stromal cells (r = -0.251; p = 0.047 and r = 0.255; p = 0.044, respectively). Also, PD-L1 expression in the stromal cells of gastric tumors directly correlates with PD-L1 expression in tumor cells and the content of all stromal markers examined. A similar pattern was observed for B cells: namely, the content of CD20+ cells in tumor stroma positively correlates with both macrophage content and PD-L1 expression in both stroma and tumor cells, and this correlation was statistically significant.

**Fig. 4:**
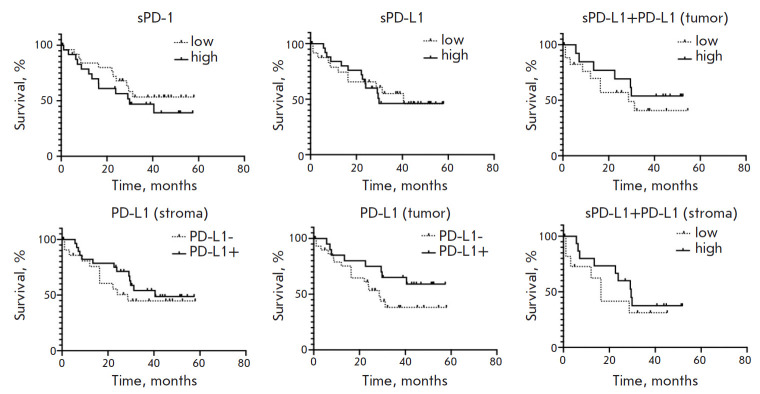
Analysis of the overall survival of GC patients depending on the content of soluble (sPD-L1, sPD-1) and tissue (PD-L1, PD-1) forms of the main components of the PD-1/PD-L1 immunity checkpoint

**Prognostic significance of PD-L1/PD-1 in cancer patients **We analyzed the prognostic significance of the markers studied and their combinations in GC patients. Depending on the content of the soluble forms of the studied proteins, patients were divided into two groups: those with a high and low content of the studied markers relative to the median. In the case of the PD-L1 tissue expression, patients were divided into two groups: depending on the presence or absence of this protein separately in tumor and stromal cells. In addition, we analyzed survival depending on the complex content of both soluble sPD-L1 and the tissue form of PD-L1. The survival plots of patients are shown in Fig. 4.

This study failed to establish a relationship between the sPD-1 and sPD-L1 levels in GC patients and the survival prognosis. For the tissue form of PD-L1, an inconsistent pattern was revealed. However, it should be noted that, for PD-L1 in tumor cells, we observed a trend toward the prognostic significance of the marker; i.e., a high expression of this protein in tumor cells of GC patients is a more favorable prognostic factor than a low expression of the marker (p = 0.122). Also, a comprehensive analysis indicated that a simultaneous high content of tissue and soluble forms of PD-L1 was not a prognostic marker in cancer.

**Fig. 5:**
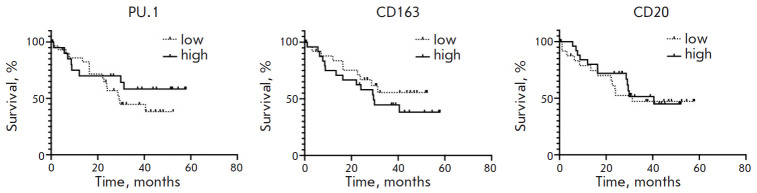
Analysis of the overall survival of GC patients depending on the PU.1, CD163, and CD20 content in the tumor stroma

Next, we analyzed the prognostic significance of PU.1, CD20, and CD163 in cancer. The results are shown in Fig. 5.

The data in Fig. 4 show that the studied stromal markers (PU.1, CD163, and CD20) are not prognostically significant in GC.

In addition, we performed a multivariate statistical analysis of the prognostic significance of all investigated markers. The results are presented in Table 4.

**Table 4:** Statistical analysis of the prognostic significance of sPD-1, sPD-L1, PD-1, PD-L1, CD20, CD163, and PU.1 in GC patients

Metrics	Univariate analysis			Multivariate analysis		
	HR	95% CI	p	HR	95% CI	p
sPD-1 (high/low)	1.443	(0.646–3.226)	0.366	0.971	(0.915–1.013)	0.234
sPD-L1 (high/low)	1.038	(0.466–2.315)	0.927	0.999	(0.988–1.008)	0.780
PD-L1 (tumor) (high/low)	0.524	(0.235–1.167)	0.122	0.480	(0.150–1.406)	0.193
PD-L1 (stroma) (high/low)	0.721	(0.316–1.644)	0.419	0.954	(0.332–2.564)	0.927
CD20 (high/low)	0.876	(0.393–1.953)	0.745	0.992	(0.965–1.016)	0.526
CD163 (high/low)	1.509	(0.677–3.361)	0.316	1.053	(1.007–1.098)	0.019*
PU.1 (high/low)	0.654	(0.292–1.466)	0.319	0.991	(0.963–1.018)	0.497

^*^Statistically significant.

Cox regression analysis revealed that a high CD163 content in cancer is an independent prognostic factor associated with decreased overall survival.

## DISCUSSION

The clinical and prognostic significance of the microenvironment of gastric tumors is being actively studied. In this work, we analyzed the content of PU.1+, CD163+, and CD20+ in the stroma of gastric tumors and evaluated their clinical and prognostic significance. In the context of solid tumors, the clinical significance of PU.1 expression was studied in patients with breast cancer and gliomas [11, 12]. Association of its expression with progression of the disease and an unfavorable prognosis were established for both tumor types. PU.1 expression has also been studied in non-small cell lung cancer (NSCLC) [13], colorectal cancer [14], and esophageal cancer [10]. One study was devoted to the study of PU.1 expression in GC, which showed that PU.1 expression is significantly elevated in gastric tumor tissue compared to the relative norm and is associated with an unfavorable prognosis and disease progression. Moreover, high PU.1 expression positively correlates with the number of activated CD4 memory T cells, resting NK cells, M2 macrophages, resting dendritic cells, and neutrophils in the tumor stroma [15]. Our study failed to reveal any prognostic significance of this protein, but consistent with the literature data, we observed a positive correlation of the PU.1+ cell content with macrophages and B-cells, as well as PD-L1+ cells in the tumor stroma.

A large number of studies are devoted to the analysis of the CD163+ macrophage content in gastric tumors, but the results are rather inconclusive. The literature suggests that CD163 expression is often associated with an unfavorable prognosis of various solid tumors [16]. However, for gastrointestinal tumors, it has been shown that CD163 can be a marker of good prognosis, particularly in esophageal cancer [17] and colorectal cancer [18]. For GC, an increased density of CD163+ macrophages in tumor stroma has been shown to be associated with the activation of the immune response and improved patient survival according to single-factor analysis [19]. However, opposite results have also been reported. A study of 148 tumor tissue samples revealed that high CD68+/CD163+ infiltration was a marker of unfavorable prognosis [20]. Other researchers demonstrated that an elevated CD163+ cell content was associated with large tumor size, low tumor differentiation, and metastases in regional lymph nodes. Moreover, the CD163 density increased with the depth of invasion, stage of the disease, and increased expression of tumor stem cell markers. The authors also found that an increased expression of this marker was associated with disease recurrence [21, 22]. The data we obtained are in agreement with the literature data; namely, a high content of CD163+ cells in the tumors of GC patients is an independent marker of an unfavorable prognosis in this pathology. There is also evidence in the litera ture that increased CD163 expression is characteristic of PD-L1+ cancer compared to PD-L1 [23]. Our results demonstrate that the CD163+ cell content in tumor stroma positively correlates with PD-L1 expression in stromal but not in tumor cells in GC.

At the next stage of the study, we analyzed the content of CD20+ cells in the stroma of the tumors of GC patients. Various studies report the presence of CD20+ B-lymphocytes in tumors of different types to have an ambiguous effect on survival prognosis and tumor stage [24]. For example, it was shown that in breast cancer, the total number of CD20+ B-lymphocytes is associated with tumor progression [25], while in some cases of ovarian, liver, and colorectal cancer, the correlation was the inverse [26, 27, 28]. The increased content of CD20+ B-lymphocytes in the stroma was shown to be associated with a better prognosis for GC patients. However, no association between the B-lymphocyte count and clinical and morphological characteristics was revealed [29]. Other researchers have demonstrated similar results, showing that a higher CD20+ B-cell density in the stroma is associated with a better prognosis. This study has also found the CD20 expression to be associated with CD68 in the tumor stroma. Interestingly, some stromal immune cells expressed Ki-67 and these were mostly CD20+ cells. Moreover, a combination of Ki-67+ and CD20+ demonstrated better prognostic potential for GC [30]. The results of our study demonstrate the lack of prognostic significance of CD20 in GC, indicating the need to use combinations of markers to improve the effectiveness of predicting the clinical course of the disease.

About two dozen studies are devoted to the prognostic significance of PD-L1 tissue expression. Most of those studies suggest an unfavorable prognostic significance of this protein expression in GC tumor cells [31]. However, some studies suggest high PD-L1 expression in tumor cells to be a good prognosis marker [32, 33]. Our study has demonstrated that PD-L1 expression in tumor cells is associated with a higher overall survival chance for patients, with no such pattern found for PD-L1 expression in stromal cells or the concentration of its soluble form in plasma.

## CONCLUSION

The results obtained in this study suggest that markers of stromal cells in gastric malignancies can potentially be used to plot treatment strategies and disease prognosis. However, current techniques, namely single-color immunohistochemistry, do not provide a sufficiently informative response. In order to use stromal markers effectively in the case of GC, the development of a comprehensive assay involving the determination of several serum markers and a multiplex analysis of several tumor stroma markers is needed.
